# Kawasaki Disease With Combined Cholestatic Hepatitis and *Mycoplasma pneumoniae* Infection: A Case Report and Literature Review

**DOI:** 10.3389/fped.2021.738215

**Published:** 2022-02-09

**Authors:** Shen-Wen Huang, Sheng-Chieh Lin, Shih-Yen Chen, Kai-Sheng Hsieh

**Affiliations:** ^1^Division of Allergy, Asthma, and Immunology, Department of Pediatrics, Shuang Ho Hospital, Taipei Medical University, Taipei, Taiwan; ^2^Department of Primary Care Medicine, Shuang Ho Hospital, Taipei Medical University, Taipei, Taiwan; ^3^Department of Pediatrics, School of Medicine, College of Medicine, Taipei Medical University, Taipei, Taiwan; ^4^Division of Pediatric Gastroenterology, Department of Pediatrics, Shuang Ho Hospital, Taipei Medical University, Taipei, Taiwan; ^5^Division of Pediatric Cardiology, Department of Pediatrics, Shuang Ho Hospital, Taipei Medical University, Taipei, Taiwan

**Keywords:** Kawasaki disease (KD), cholestatic hepatitis, jaundice, abdominal pain, *Mycoplasma pneumoniae* infection

## Abstract

Kawasaki disease (KD), also called mucocutaneous lymph node syndrome, is a febrile multisystem vasculitis mainly affecting children younger than 5 years. KD typically manifests as skin lesions and in the lymph nodes and oral and conjunctival mucosa. It may induce coronary artery abnormalities, such as aneurysms, but gastrointestinal and hepatobiliary involvement are not common. We reviewed 32 cases of patients with a diagnosis of KD with hepatobiliary involvement between 2000 and 2021 and present the case of a 4-year-old girl who received a diagnosis of KD with combined cholestatic hepatitis and *Mycoplasma pneumoniae* infection. In the 33 cases reviewed, in addition to the classical clinical findings of KD, the most common clinical presentations were jaundice and abdominal pain. Moreover, abnormal laboratory results indicating hyperbilirubinemia, cholestasis, and hepatitis, among other conditions, were noted. Abdominal ultrasonography revealed abnormal findings in more than half children with KD with hepatobiliary involvement. Furthermore, cardiac involvement was noted in a high proportion of the patients. In particular, we noted the case of a 4-year-old girl with a rare presentation of 3-day fever combined with abdominal pain and jaundice. Her levels of aspartate aminotransferase, alanine aminotransferase, total bilirubin, direct bilirubin, alkaline phosphatase, and γ-glutamyl transpeptidase were 489 (15–50) U/L, 253 (5–45) U/L, 4.3 (<1.5) mg/dl, 2.4 (<0.2) mg/dl, 337 (134–315) U/L, and 145 (5–32) U/L, respectively. These results were indicative of cholestatic hepatitis. Furthermore, her serological test results for mycoplasma infection were positive. KD was diagnosed because the patient had high fever for more than 5 days and presented with lymphadenopathy on the left side of neck, a polymorphic skin rash, redness of oral mucosa with strawberry tongue, and nonpurulent conjunctival congestion. After intravenous immunoglobulin injection (IVIG) and acetylsalicylic acid administration, the fever subsided rapidly and clinical manifestations, such as jaundice and abdominal pain, were mitigated. The laboratory parameters gradually returned to within normal ranges. Echocardiography revealed no aneurysm. In conclusion, KD with cholestatic hepatitis should be considered when pediatric patients present with fever combined with abdominal pain and jaundice. Early treatment with IVIG and aspirin is recommended and can effectively relieve cholestatic hepatitis.

## Introduction

Kawasaki disease (KD) is a multisystem inflammatory disease encompassing medium vessel vasculitis, potentially including the coronary arteries. The prevalence of KD among children in Japan between 1994 and 2002 and Taiwan between 1997 and 2010 was 119.6–151.2 and 48.5–82.8 per 100,000 person-years, respectively (1, 2). The average male–female ratio of KD was 4:1, with onset before the age of 5 years in 80% of cases (1). The etiology of KD remains unclear; one study reported immunologic abnormalities, but further investigation is required (3). KD diagnosis is based on various clinical measures provided by the American Heart Association and Japanese Ministry of Health (4, 5). Typically, KD symptoms include fever lasting at least 5 days combined with at least four of the following five manifestations: (i) conjunctival congestion in both eyes; (ii) changes in the lips and oral cavity, such as reddening of the lips, strawberry tongue, or diffuse injection of oral and pharyngeal mucosa; (iii) polymorphous exanthema; (iv) changes in the peripheral extremities; and (v) acute nonpurulent cervical lymphadenopathy and the exclusion of other diseases with similar findings (1). Alternatively, patients may present with three or fewer of these criteria along with coronary abnormalities or abnormal laboratory data, and this condition is classified as incomplete KD (4–6). Gastrointestinal and hepatobiliary involvement are rare clinical presentations in KD (7), and respiratory, neurological, genitourinary, and musculoskeletal involvement are also relatively uncommon clinical manifestations (1). The most common reason for sudden death in KD is cardiovascular involvement (1), but the administration of oral aspirin and intravenous immunoglobulin (IVIG) within 10 days of symptom onset can reduce the risk of coronary abnormalities (6).

Herein, we report the case of a girl presenting with an unusual initial onset of KD characterized by fever, acute abdominal pain, jaundice, and lymphadenopathy on the left side of the neck. This patient received early treatment with aspirin and IVIG. We also review 32 other cases of KD with hepatobiliary involvement reported between 2000 and 2021; thus, a total of 33 cases, including our case, were reviewed.

## Method

We conducted a literature search for relevant articles published in English between January 1, 2000, and November 1, 2021, in PubMed, Google Scholar, Crossref, and Web of Science by using the following keywords: (Kawasaki disease) AND (jaundice), (Kawasaki disease) AND (cholestasis), (Kawasaki disease) AND (cholestatic), (Kawasaki disease) AND (hepatitis). An article was excluded if no laboratory hepatobiliary data or intact image report were available or the patient was older than 18 years. Initially, we included 26 articles plus the article on the case reported herein, but three articles were then excluded. Finally, we reviewed 24 articles involving 33 cases (Table 1).

**Table 1:** Flowchart diagram of data collection.

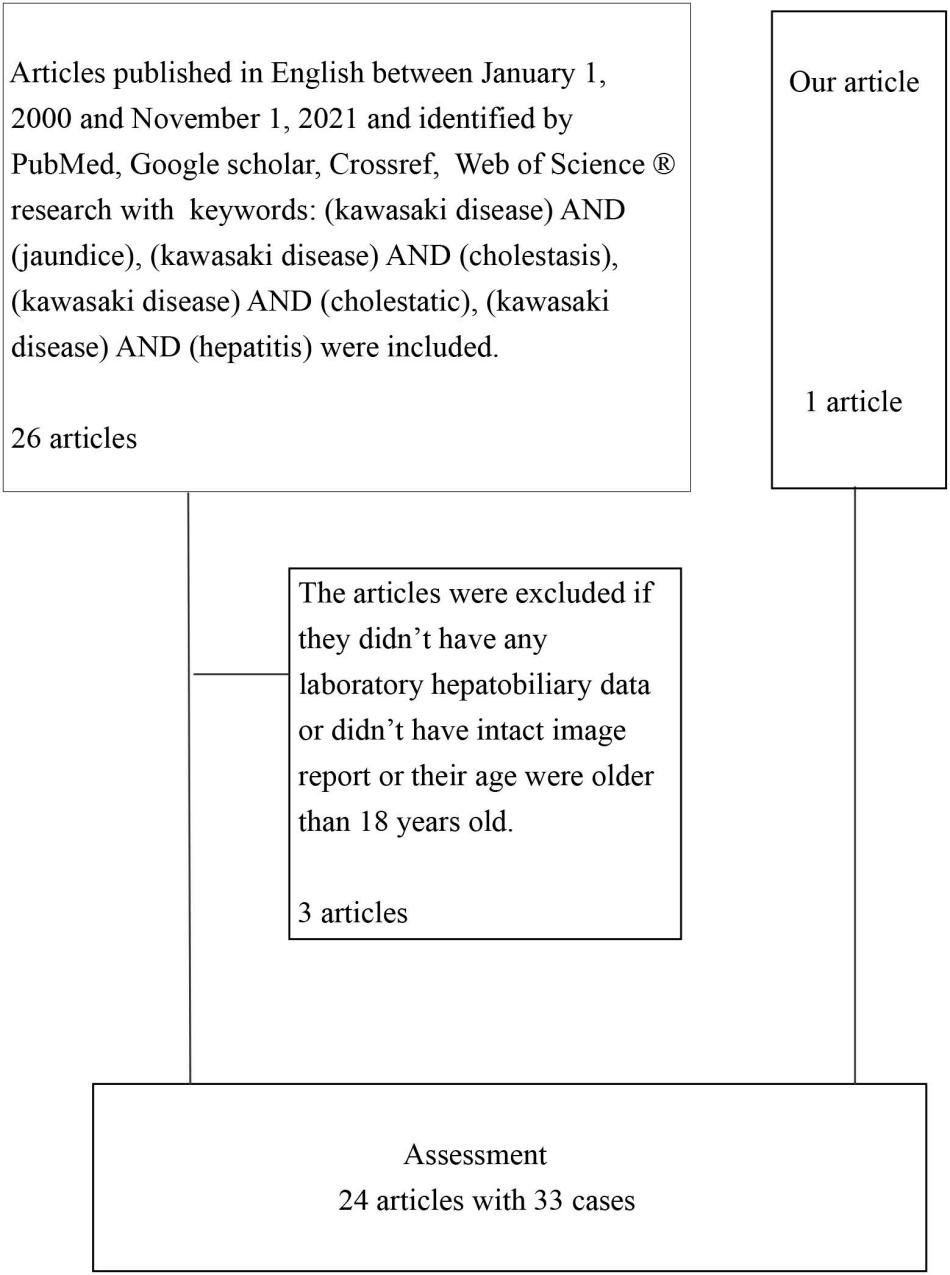

### Case Description

The patient was a girl aged 4 years and 2 months who was otherwise healthy before presenting with KD symptoms; she had achieved normal developmental milestones and received regular vaccinations. She did not have any relevant medical, psychosocial, or family history. She was admitted to our hospital because she had persistent high fever (≤ 38.5°C) for 3 days. Her parents stated that she had first developed diffuse abdominal pain and swelling on the left side of the neck and had initially been taken to a local general practitioner, who suspected acute gastroenterocolitis. Because of the persistent fever and subsequent development of jaundice, she was brought to our hospital for further evaluation. When admitted, she had poor appetite and vomiting and had developed symptoms of respiratory tract infection, such as rhinorrhea and coughing with sputum. Sore throat, dyspnea, diarrhea, constipation, dysuria, and reductions in urine output were not noted.

**Figure 1:**
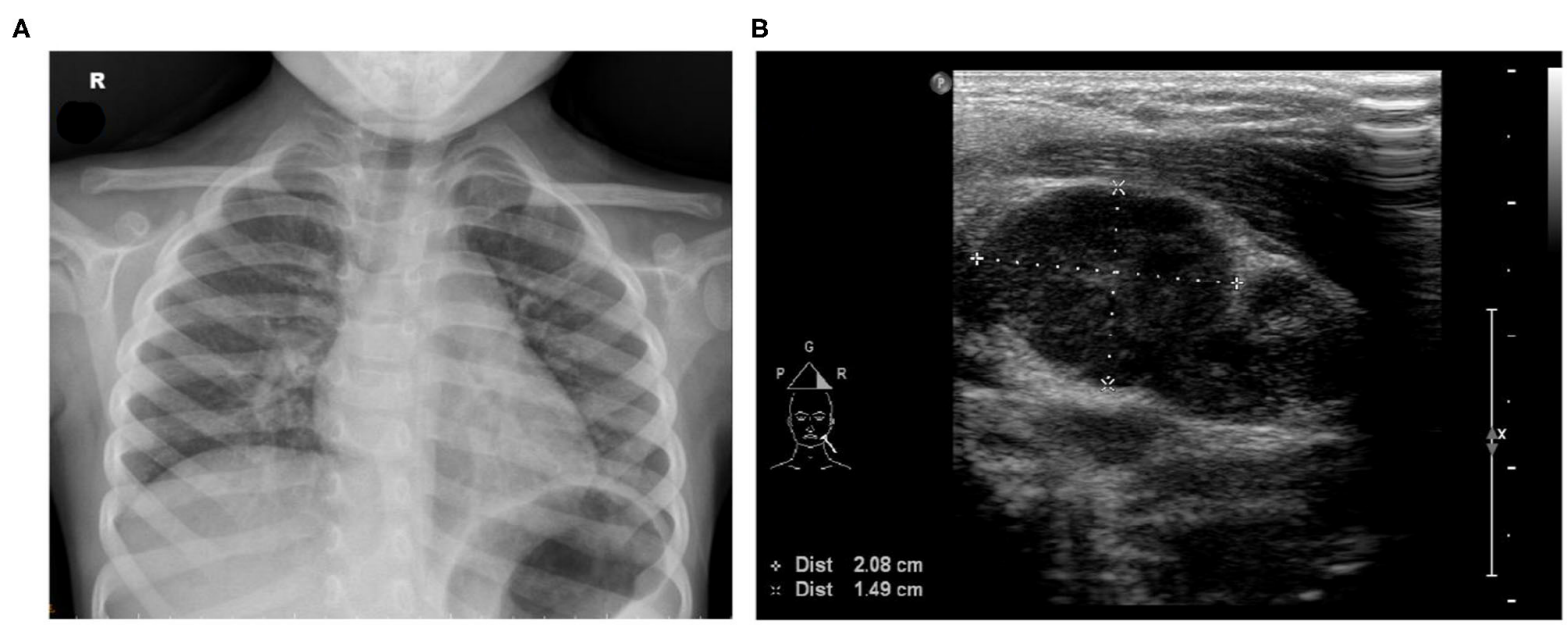
**(A)** Chest X-ray image exhibiting increased bronchoalveolar infiltration over the right lung field. **(B)** Neck tissue echography revealing multiple lymphadenopathy on the left side of the neck (largest dimensions: 2.08 × 1.49 cm).

A physical examination revealed that the patient had a 39°C fever, scleral icterus in both eyes, nonpurulent conjunctival congestion, oral mucosa redness, swelling on the left side of the neck, diffuse abdominal tenderness in the periumbilical area, and crackles in the lungs. Chest X-ray revealed an elevated level of pulmonary infiltrates in both lungs (Figure 1A). Her laboratory data were as follows: hemoglobin = 10.2 (normal range = 11.0–14.5) g/dl, leukocytes = 24,700 (4,000–12,000) 10^6^/L, thrombocytes = 250,100 (130,000–400,000) 10^6^/L, C-reactive protein (CRP) = 14.17 (<1) mg/dl, erythrocyte sedimentation rate = 95 (0–20) mm/h, prothrombin time = 17.3 (11.0–14.5) s, international normalized ratio = 1.32 (<1.20), activated partial thromboplastin time = 45.8 (32.0–45.0) s, aspartate aminotransferase = 489 (15–50) U/L, alanine aminotransferase = 253 (5–45) U/L, total bilirubin = 4.3 (<1.5) mg/dl, direct bilirubin = 2.4 (<0.2) mg/dl, alkaline phosphatase = 337 (134–315) U/L, γ-glutamyl transpeptidase = 145 (5–32) U/L, amylase = 112 (26–115) U/L, lipase = 58 (22–51) U/L, blood urea nitrogen = 11 (5–18) mg/dl, serum creatinine = 0.49 (0.20–0.60) mg/dl, albumin = 3.9 (3.5–5.6) g/dl, ceruloplasmin = 24.2 (20–60) mg/dl, and Cu = 860 (700–1,500) μg/L. Her serological test results revealed positivity for *Mycoplasma pneumoniae* [*M. pneumoniae*; immunoglobulin G = 1,810.79 (<100.00) 10^3^U/L and immunoglobulin M = 3,381.64 (<770.00) 10^3^U/L] but negativity for Epstein–Barr virus, cytomegalovirus, and hepatitis A, B, and C. Furthermore, tests for antinuclear antibodies, rheumatoid factor, antineutrophil cytoplasmic antibodies, and antistreptolysin O were negative, as were the results of the influenza A/B and group A streptococcus antigen screening test and pneumococcal urinary antigen testing. Nasal swab and stool tests revealed negative results for adenovirus, and results for rotavirus were also negative. The stool microscopy results were normal. Samples were collected for culture, and the patient was treated with 80 mg/kg ceftriaxone for enteric fever and cholestatic hepatitis and with azithromycin for bronchopneumonia (induced by the *M. pneumoniae* infection). Treatment response was poor, and the fever persisted. Abdominal ultrasonography indicated acute gastroenterocolitis with ileus and pelvic ascites but without cystic duct or bile duct dilatation. Neck tissue ultrasonography revealed multiple lymphadenopathies on the left side of the neck (largest dimensions: 2.08 cm × 1.49 cm; Figure 1B).

The high fever persisted for 2 days after admission (>5 days total), and a polymorphic skin rash developed, accompanied by lymphadenopathy on the left side of the neck, oral mucosa redness with strawberry tongue, and nonpurulent conjunctival congestion. The patient received a diagnosis of KD, and a single dose of IVIG as a 12-h 2 g/kg intravenous infusion and acetylsalicylic acid (50 mg/kg/day, divided into four doses) was administered. Echocardiography revealed no coronary artery involvement. After the administration of IVIG and aspirin, the fever and abdominal pain subsided and the jaundice and skin rash gradually resolved. Acetylsalicylic acid was reduced to one dose of 3–5 mg/kg at 48 h after the fever had subsided, and liver function improved gradually. Further laboratory examination results are presented in Table 2. The patient was discharged and followed up at our outpatient department. Abnormal laboratory data gradually returned to normal, and the echocardiography results for the coronary arteries in the outpatient department follow-up were normal.

**Table 2:** Laboratory data obtained during admission and follow-up.

	**Admission**	**In hospital**	**In hospital**	**In hospital**	**OPD f/u**	**OPD f/u**
**Time**	**Day1**	**Day3**	**Day4**	**Day7**	**Day24**	**Day50**
Therapeutic intervention		IVIG (IVD) + ASA (PO)	ASA (PO)	ASA (PO)	ASA (PO)	ASA (PO)
Laboratory parameters						
WBCs (10^6^/L) (4.00–12.00)	24.70 × 10^3^		22.00 × 10^3^	11.50 × 10^3^		5.70 × 10^3^
Neutrophil (%) (40.0–74.0)	93.0		79.5	48.9		
Lymphocyte (%) (19.0–48.0)	2.5		13.5	37.3		
Monocyte (%) (3.4–9.0)	4.2		3.7	7.6		
Eosinophil (%) (0.0–7.0)	0.1		3.0	5.4		
Basophil % (0.0–1.5)	0.1		0.4	0.9		
RBCs (10^6^/L) (4.20–5.40)	3.52 × 10^6^		3.25 × 10^6^	3.32 × 10^6^		4.02 × 10^6^
HGB (g/dl) (11.0–14.5)	10.2		9.4	9.6		11.3
Platelets (10^6^/L) (130–400)	251 × 10^3^		327 × 10^3^	343 × 10^3^		305 × 10^3^
PT (s) (11.0–14.5)	17.3		15.1	13.9	13.6	
PT INR (<1.20)	1.32		1.14	1.05	1.00	
MNPT (s)	13.3		13.3	13.3	13.6	
APTT (s) (32.0–45.1)	45.8		56.1	48.5	44.6	
ESR (mm/1hr) (0–20)	95					
CRP (mg/dl) (<1.00)	14.17			6.33	0.65	
GOT (U/L) (15–59)	489			43	29	
GPT (U/L) (5–45)	253			74	13	
ALK-P (U/L) (134–315)	337				202	
r-GT (U/L) (5–32)	145				13	
Bilirubin D (mg/dl) (<0.2)	2.4			0.3	0.2	0.1
Bilirubin T (mg/dl) (<1.5)	4.3			1.1	0.4	
Albumin (g/dl) (3.5–5.6)	3.9					
Lipase (U/L) (22–51)	58				29	
Amylase U/L (26–115)	112					
Cu (ug/L) (700–1,500]	860					
Ceruloplasmin (mg/dl) (20.0–60.0)	24.2					
BUN (mg/dl) (5–18)	11					
Creatinine (mg/dl) (0.20–0.60)	0.49					

*IVIG, intravenous immunoglobulin; ASA, acetylsalicylic acid; OPD, outpatient department; IVD, intravenous drip; PO, oral; WBC, white blood cell; RBC, red blood cell; HGB, Hemoglobin; PT, Prothrombin time; APTT, activated partial thromboplastin time; ESR, erythrocyte sedimentation rate; CRP, C-Reactive protein; GOT, glutamic-oxalocetic transaminase; GPT, glutamic-pyruvic transaminase; ALK-P; alkaline phosphatase; r-GT, γ-glutamyltransferase; BUN, blood urea nitrogen*.

## Discussion

In KD, hepatobiliary involvement is an atypical presentation. Our patient was a girl aged 4 years and 2 months with KD. In addition to fever, a classical symptom of KD, our patient had lymphadenopathy on the left side of the neck, a polymorphic skin rash, redness of the oral mucosa with strawberry tongue, and nonpurulent conjunctival congestion. Echocardiography indicated no coronary artery involvement. Jaundice with abdominal pain was diagnosed early, and febrile cholestatic hepatitis was also noted. Studies have reported different rates of diarrhea, vomiting, abdominal pain, hepatic dysfunction, and bile duct hydrops as nonspecific symptoms of KD, although these symptoms are not among the diagnostic criteria (4, 8, 9). In the present case, we observed the development of cholestatic hepatitis. Our findings indicate that cholestatic hepatitis can be an atypical early sign of KD, and early treatment with IVIG can rapidly improve liver function, cholestasis, and other conditions and also prevent cardiac complications.

In this study, in addition to the reported case, we reviewed another 32 cases of KD with hepatobiliary involvement reported between 2000 and 2021 in studies retrieved from PubMed, Google Scholar, Crossref, and Web of Science; the details of these studies are presented in Tables 3, 4 (7, 9–30). The mean age of the pediatric patients in the reports was 5.26 years. The age at the onset of KD with hepatobiliary involvement was later than the average age at KD onset, with 73 and 27% patients presenting with classical and incomplete KD, respectively. In addition to the classical clinical findings, the most common clinical presentations in these patients were jaundice and abdominal pain. In total, 76% of the patients presented with jaundice and 48% presented with abdominal pain. Laboratory data indicated hyperbilirubinemia, cholestasis, hepatitis, and other abnormalities. For five patients, direct bilirubin data were not available, and for two, total bilirubin data were not available; however, all the other patients exhibited an increase in direct bilirubin and total bilirubin. Furthermore, all the patients except three (for whom serum inflammatory marker data were not available) exhibited an increase in serum inflammatory markers. In total, 91% of the patients had increased levels of liver enzymes. However, γ-glutamyl transpeptidase activity was examined in 24 patients, all of whom had high cholestatic indexes. The 33 patients were all prescribed IVIG and oral acetylsalicylic acid when KD was diagnosed. After treatment, fever subsidence was noted for all these patients, and rapid regression of jaundice was noted in 96% of these patients. Although jaundice was identified in most of the cases, not all had notable results for abdominal ultrasonography and echocardiography. Moreover, 33% of the patients had an enlarged liver, 18% had a thickened gallbladder wall, and one patient was revealed to have intrahepatic bile duct stasis through abdominal ultrasonography. Despite the diverse abdominal ultrasonography results, only one of the patients had autoimmune sclerosing cholangitis; the others all had negative abdominal ultrasonography results after follow-up. In addition, 39% of the patients had dilated coronary arteries, resulting in aneurysms. Preventing coronary artery aneurysm is crucial because it may cause stenosis of the vessels, often resulting in coronary artery obstruction and myocardial ischemia (1). Furthermore, 30% of the patients had other comorbidities. Overall, abnormal abdominal ultrasonography findings were obtained for 61% of the patients, and echocardiography revealed cardiac involvement in 45% of the patients. A high proportion of children with KD with hepatobiliary involvement appear to have cardiac involvement, but the physiopathology of hyperbilirubinemia and cholestatic liver damage in KD remains undetermined. Vasculitis-associated inflammation and obstruction in the liver and gallbladder are considered the cause of increased transaminase levels and cholestasis (9). Increased serum γ-glutamyl transpeptidase activity is a marker of cholangiocyte injury and is indicative of bile duct damage. Acute hydrops of the gallbladder in some patients suggests that inflammatory cholangiocyte injury may extend from small to large bile ducts (17). However, hydrops of the gallbladder is an atypical sign of KD (1). Children with KD develop isolated gall bladder hydrops without bilirubin or liver transaminase elevation, which should also be considered. Abdominal ultrasonography in 91% of the patients reviewed did not indicate hydrops of the gallbladder, and cholestasis in 96% of these patients was mitigated after IVIG treatment.

**Table 3:** Clinical characteristics of 33 patients with KD and hepatobiliary involvement.

**References**		**This study**	**Anjani et al. (10)**	**Morita et al. (11)**	**Menon et al. (12)**	**Paglia et al. (13)**	**Sarkar et al. (14)**	**Bylund et al. (15)**	**Vázquez et al. (16)**	**Kiliç et al. (9)**	**Koca et al. (17)**	**Goknar et al. (18)**
Type of Kawasaki disease		Classical	Classical	Classical	Classical	Classical	Classical	Classical	Classical	Incomplete	Classical	Classical
Age		4 yr	3 yr	2 yr	7 yr	7 month	4 yr	6 yr	9 yr	7 yr	9 yr	3 yr
Symptoms	Days of fever before diagnosis	5 d	20 d	4 d	10 d	4 d	6 d	8 d	5 d	5 d	9 d	9 d
	Abdominal pain	+	-	-	-	-	+	-	-	+	-	-
	Conjunctival injection	+	+	+	+	+	+	+	+	+	+	+
	Changes in extremities	-	+	+	+	+	-	+	+	-	+	+
	Rash	+	+	+	+	+	+	+	+	+	+	+
	Cervical lymphadenopathy	+	-	+	-	+	+	+	+	-	+	-
	Changes lips/oral cavity	+	+	+	+	+	+	+	+	+	+	+
	Jaundice	+	+	-	-	-	+	+	+	-	+	+
Laboratory examination	Hemoglobin (g/dl)	10.2	8.2	Not available	8.6	Not available	10	Not available	Not available	13	12	11.4
	Leukocytes (10^6^/L)	24,700	23,300	16,400	11,700	Not available	17,500	16,300	27,330	15,700	35,000	24,300
	Platelets (10^6^/L)	251,000	886,000	Not available	265,000	Not available	494,000	369,000	364,000	93,000	230,000	360,000
	CRP (mg/dl)/ESR (mm/1 hr)	14.17/95	3.1/Not available	15.7/50	7.4/Not available	Not available	3.5/113	6.5/57	17.1/75		21.1/69	20.9/Not available
	AST/ALT (U/L)	489/253	215/215	5,323/1,554	100/175	186/240	159/260	59/169	80/60	109/202	159/211	149/150
	Bilirubin, total/direct (mg/dl)	4.3/2.4	8.9/6.8	1.5/Not available	1.9/Not available	Not available	5.2/4.3	5.4/2.9	7.1/5.4	3.49/3.42	8.2/5.6	7.4/6.15
	ALK-P/r-GT (U/L)	337/145	Not available	Not available/149	Not available	Not available	530/270	425/241	Not available	Not available	Not available/151	Not available/310
Comorbidities		Mycoplasma infection	1. Grade 2 Hepatic Encephalopathy 2. Macrophage activation syndrome	Bronchitis			-	-	Acute kidney injury	Acute cholangitis/ cholecystitis	-	Arthritis
**References**		**Kaman et al**. **(****19****)**		**Keeling et al**. **(****20****)**		**Rosencrantz et al**. **(****21****)**	**Perera et al**. **(****22****)**	**Talebian et al**. **(****23****)**	**Grewal et al**. **(****24****)**	**Jafari et al**. **(****25****)**	**Karpathios et al**. **(****26****)**	
		**Case 1**	**Case 2**	**Case 1**	**Case 2**						**Case 1**	**Case 2**
Type of Kawasaki disease		Incomplete	Incomplete	Classical	Incomplete	Classical	Incomplete	Classical	Classical	Incomplete	Classical	Classical
Age		6 yr	2.5 yr	12 yr	6 yr	4.5 yr	11 yr	4 yr	7 yr	23 month	7 yr	3.5 yr
Symptoms	Days of fever before diagnosis	9 d	9 d	6 d	10 d	7 d	7 d	9 d	12 d	10 d	5 d	6 d
	Abdominal pain	+	-	+	+	+	+	+	+	+	+	-
	Conjunctival injection	+	-	+	+	+	+	+	+	+	+	+
	Changes in extremities	+	-	+	-	+	-	+	+	+	+	+
	Rash	-	-	-	-	+	-	+	+	+	+	+
	Cervical lymphadenopathy	-	-	+	+	+	+	+	+	-	+	-
	Changes lips/oral cavity	+	-	+	+	+	-	+	+	-	+	+
	Jaundice	+	-	+	-	-	+	+	+	+	+	+
Laboratory examination	Hemoglobin (g/dl)	9.1	10.1	Not available	Not available	Not available	Not available	10	Not available	7.9	13.7	11
	Leukocytes (10^6^/L)	8,700	22,400	15,400	21,800	Not available	152,000	17,500	9,100	7,100	11,920	31,400
	Platelets (10^6^/L)	182,000	413,000	451,000	746,000	Not available	250,000	494,000	840,000	396,000	264,000	346,000
	CRP (mg/dl)/ESR (mm/1hr)	16/55	36/145	15/Not available	Not available	Not available	9.6/120	18/64	Not available/49	9/135	8/Not available	24.7/ Not available
	AST/ALT (U/L)	29/42	44/51	17/42	26/23	115/146	Not available	159/260	90/84	177/198	84/138	41/124
	Bilirubin, total/direct (mg/dl)	4.8/4	5.4/4.1	5.37/2.86	Not available	0.7/Not available	3.9/3.0	5.2/4.3	11.7/10.5	5.7/2.6	7.45/4.86	15.1/12.5
	ALK-P/r-GT (U/L)	Not available/168	Not available/444	42/50	Not available	708/655	Not available	530/270	Not available	2,786/392	316/134	186/56
Comorbidities		-		-	recurrent Incomplete KD	autoimmune sclerosing cholangitis	-	-	-	-	Gilbert syndrome	Gilbert syndrome
**References**		**Ibáñez-Alcalde et al**. **(****27****)**			**Taddio et al**. **(****28****)**					**Valentini et al**. **(****7****)**	**Grech et al**. **(****29****)**	**Chen et al**. **(****30****)**
		**Case 1**	**Case 2**	**Case 3**	**Case 1**	**Case 2**	**Case 3**	**Case 4**	**Case 5**			
Type of Kawasaki disease		Classical	Classical	Classical	Classical	Incomplete	Classical	Incomplete	Incomplete	Classical	Classical	Classical
Age		4 yr	17 month	18 month	3 yr	10 yr	4 yr	1 yr	8 yr	6 yr	3.5 yr	10 yr
Symptoms	Days of fever before diagnosis	4 d	7 d	4 d	5 d	20 d	10 d	5 d	6 d	9 d	7 d	13 d
	Abdominal pain	unknown	unknown	unknown	-	-	-	-	+	+	+	+
	Conjunctival injection	+	-	+	+	-	+	+	+	+	+	+
	Changes in extremities	+	+	+	-	+	+	-	-	+	+	+
	Rash	+	+	+	+	+	+	+	+	+	+	+
	Cervical lymphadenopathy	+	+	+	+	-	-	-	-	+	-	-
	Changes lips/oral cavity	+	+	+	+	+	+	+	+	+	+	+
	Jaundice	+	+	+	+	+	+	+	+	-	+	+
Laboratory examination	Hemoglobin (g/dl)	11.8	11.1	12.4	Not available	Not available	Not available	Not available	Not available	10.8	11.7	13.5
	Leukocytes (10^6^/L)	16,100	8,560	15,250	13,400	15,950	13,750	14,100	12,000	19,220	297,000	14,700
	Platelets (10^6^/L)	397,000	604,000	416,000	Not available	Not available	Not available	Not available	Not available	normal	448,000	normal
	CRP (mg/dl)/ ESR (mm/ 1 hr)	4.57/ 76	1.79/85	12.04/86	5.4/49	4.4/50	3.3/77	2.7/62	2/52	23.2/ Not available	29/ Not available	15.2
	AST/ALT (U/L)	106/ 160	235/116	518/458	160/210	1,080/1,480	1,100/1,500	200/800	100/440	534/548	135/99	145/202
	Bilirubin, total/direct (mg/dl)	4.5/ 3.98	5.43/4.14	3.54/3.33	4/3.1	5.2/5	3.4/2.5	6/4.9	3.1/1.7	7.1/4	13.6/ Not available	4.2/2.4
	ALK-P/r-GT (U/L)	385/179	967/655	157/94	Not available/130	not showed/160	not showed/155	Not available/140	Not available/160	Not available/272	738/180	Not available
Comorbidities		-	-	-	-	-	-	-	-	-	-	-

**Table 4:** Imaging findings for 33 patients with KD and hepatobiliary involvement.

**References**		**Abdominal ultrasonography**	**Echocardiography**
This study		Ileus and pelvic ascites	No cardiac involvement
Anjani et al. (10)		Hepatomegaly	Left main coronary artery dilatation
Morita et al. (11)		Thickening of the gallbladder wall, dilatation of the CBD	Slight dilatation of the left main trunk
Menon et al. (12)		Unremarkable	No cardiac involvement
Paglia et al. (13)		Mild hepatomegaly	No cardiac involvement
Sarkar et al. (14)		Mild hepatomegaly	No cardiac involvement
Bylund et al. (15)		Unremarkable	No cardiac involvement
Vázquez et al. (16)		Unremarkable	Aneurysm in the right coronary artery of 5.8 mm and 8.3 mm in the left coronary artery, signs of ischemia in the side wall of the myocardium.
Kiliç et al. (9)		Acute cholangitis/cholecystitis:thickening of the gallbladder wall, hydrops, and intrahepatic bile duct stasis	Minimal pericardial effusion, and mild mitral and tricuspid regurgitation in the left ventricle.
Koca et al. (17)		Unremarkable	Fusiform dilatation in the right coronary artery
Kaman et al. (19)	Case 1	Subhepatic and pelvic fluid	Bilateral diffuse dilatation of the coronary arteries (3.3 mm left and 3.6 mm right) and minimal mitral valve insufficiency
	Case 2	Unremarkable	Irregularity and dilatation of left CA ostia and mitral valve insufficiency
Goknar et al. (18)		Unremarkable	Coronary artery dilatation
Keeling et al. (20)	Case 1	Hepatomegaly	No cardiac involvement
	Case 2	Hepatomegaly	No cardiac involvement
Rosencrantz et al. (21)		Gallbladder hydrops and nonobstructive intrahepatic and extrahepatic biliary ductal dilatation	No cardiac involvement
Perera et al. (22)		Hydrops of the gallbladder	8.1 mm giant aneurysm in LAD and a 6 mm sized aneurysm in the RCA. Both were proximal and fusiform.
Talebian et al. (23)		Mild hepatomegaly	No cardiac involvement
Grewal et al. (24)		Enlarged liver (spanning 12.7 cm)	Dilated right coronary artery distally (4.2 mm).
Jafari et al. (25)		Hepatosplenomegaly	Three vessels aneurysms (LAD, LCA, RCA)
Ibáñez-Alcalde et al. (27)	Case 1	Mesenteric nodes >1 cm	No cardiac involvement
	Case 2	Unremarkable	No cardiac involvement
	Case 3	Edema of the bowel wall	Mitral insufficiency
Karpathios et al. (26)	Case 1	Unremarkable	No cardiac involvement
	Case 2	Mild hepatomegaly	Dilation of the right coronary artery
Taddio et al. (28)	Case 1	Unremarkable	No cardiac involvement
	Case 2	Thickening of gallbladder walls.	No cardiac involvement
	Case 3	Unremarkable	No cardiac involvement
	Case 4	Unremarkable	No cardiac involvement
	Case 5	Unremarkable	No cardiac involvement
Valentini et al. (7)		Enlarged liver, enlarged lymph-nodes, enlarged and thickened gallbladder, mild ascites and multiple bowel air–fluid levels.	Diffuse dilated and hyperechogic coronary arteries
Grech et al. (29)		Hepatomegaly	No cardiac involvement
Chen et al. (30)		Unremarkable	Dilatation of the left and right coronary arteries

Regarding the pediatric diagnosis of KD, a differential diagnosis may be required given that KD is diagnosed on the basis of clinical criteria; no specific diagnostic test has been developed. Various organ systems are involved in atypical KD, including the central nervous system and ocular, renal, joint, and gastrointestinal systems. Abdominal manifestations are sometimes acute, and severe abdominal pain similar to that associated with appendicitis or pancreatitis may lead to a surgical intervention (28). Such atypical manifestations of KD may delay treatment. Prompt KD diagnosis is crucial because IVIG administration within 10 days of the onset of fever results in a lower rate of coronary abnormalities and lowering of the risk of coronary artery aneurysm formation from 20–25% to 3–5% in those who are appropriately treated (9, 31, 32).

The etiology of KD remains unclear. Immunological responses are reported to be associated with KD, but the immunological profile associated with KD has yet to be defined (1). Furthermore, an infection-induced immunologic response may be considered a predisposing factor; according to some case reports, KD may be associated with *M. pneumoniae* infection (33–35). *M. pneumoniae* has several arrays for immunomodulation, including T cell and B cell activation and cytokine oversecretion, which are thought to trigger KD (36). *M. pneumoniae* infection can have a wide spectrum of extrapulmonary manifestations (including dermatological) that may mimic KD (37). A study reported that acute hepatitis can be included in the heterogeneous group of extrapulmonary diseases related to *M. pneumoniae* infection (38). The pathogenesis of hepatitis related to *M. pneumoniae* infection is likely to be immune mediated (38), but the clinical manifestations of KD, including strawberry tongue and jaundice, are not a clinical extrapulmonary manifestation of *M. pneumoniae* infection (1, 37). *M. pneumoniae* infection may be a trigger for KD leading to hepatobiliary problems. The exact pathological mechanisms and whether *M. pneumoniae* infection is related to KD warrant further investigation. Notably, KD combined with *M. pneumoniae* infection may present as a more severe clinical condition; thus, early treatment of the infection is essential.

This study has limitations. Not all the cases that we reviewed had complete hepatobiliary data. The child in the reported case was not tested for hepatitis E, and complete work-up for autoimmune hepatitis was not performed. However, the presence of these conditions was unlikely.

Jaundice is a critical differential diagnosis of KD, a disease requiring prompt treatment with IVIG and aspirin to alleviate symptoms and prevent fatal cardiac complications. Even if coronary abnormalities are not initially observed, cardiac involvement is highly likely in patients with KD and hepatobiliary involvement and must be treated without delay. Physicians should consider the presence of this condition when examining children presenting with febrile cholestatic hepatitis.

## Data Availability Statement

The original contributions presented in the study are included in the article/supplementary material, further inquiries can be directed to the corresponding author.

## Ethics Statement

The studies involving human participants were reviewed and approved by Taipei Medical University-Joint Institutional Review Board. Written informed consent to participate in this study was provided by the participants' legal guardian/next of kin. Written informed consent was obtained from the minor(s)' legal guardian/next of kin for the publication of any potentially identifiable images or data included in this article.

## Author Contributions

S-WH cared the patient, drafted the initial manuscript, and approved the final manuscript as submitted. S-CL diagnosed, cared and treated the patient, also conceived, drafted, reviewed, and revised the manuscript, and approved the final manuscript as submitted. S-YC and K-SH are consultants and approved the final manuscript as submitted. All authors approved the final manuscript as submitted and agree to be accountable for all aspects of the work.

## Conflict of Interest

The authors declare that the research was conducted in the absence of any commercial or financial relationships that could be construed as a potential conflict of interest.

## Publisher's Note

All claims expressed in this article are solely those of the authors and do not necessarily represent those of their affiliated organizations, or those of the publisher, the editors and the reviewers. Any product that may be evaluated in this article, or claim that may be made by its manufacturer, is not guaranteed or endorsed by the publisher.

## Abbreviations

KD: Kawasaki disease
IVIG: intravenous immunoglobulin.

## References

[1] Kawasaki T. Kawasaki disease. Proc Jpn Acad Ser B Phys Biol Sci. (2006) 82:59–71. 10.2183/pjab.82.59 25792773 PMC4323050

[2] Lin MC Lai MS Jan SL Fu YC. Epidemiologic features of Kawasaki disease in acute stages in Taiwan, 1997–2010: Effect of different case definitions in claims data analysis. J Chin Med Assoc. (2015) 78:121–6. 10.1016/j.jcma.2014.03.009 25636582 PMC7105041

[3] Matsubara T Ichiyama T Furukawa S. Immunological profile of peripheral blood lymphocytes and monocytes/macrophages in Kawasaki disease. Clin Exp Immunol. (2005) 141:381–7. 10.1111/j.1365-2249.2005.02821.x 16045726 PMC1809464

[4] Singh S Jindal AK Pilania RK. Diagnosis of Kawasaki disease. Int J Rheum Dis. (2018) 21:36–44. 10.1111/1756-185X.13224 29131549 PMC7159575

[5] Jindal AK Pilania RK Prithvi A Guleria S Singh S. Kawasaki disease: characteristics, diagnosis, and unusual presentations. Expert Rev Clin Immunol. (2019) 15:1089–104. 10.1080/1744666X.2019.1659726 31456443

[6] McCrindle BW Rowley AH Newburger JW Burns JC Bolger AF Gewitz M. Diagnosis, treatment, and long-term management of Kawasaki disease: a statement for health professionals from the Committee on Rheumatic Fever, Endocarditis and Kawasaki Disease, Council on Cardiovascular Disease in the Young, American Heart Association. Circulation. (2004) 110:2747–71. 10.1161/01.CIR.0000145143.19711.78 15505111

[7] Valentini P Ausili E Schiavino A Angelone DF Focarelli B Rosa GD. Acute cholestasis: atypical onset of Kawasaki disease. Dig Liver Dis. (2008) 40:582–4. 10.1016/j.dld.2007.10.010 18055284

[8] Baker AL Lu M Minich LL Atz AM Klein GL Korsin R. Associated symptoms in the ten days before diagnosis of Kawasaki disease. J Pediatr. (2009) 154:592–5.e2. 10.1016/j.jpeds.2008.10.006 19038400 PMC2745188

[9] Kiliç BO Baysun S Gökşen TC Akinbingöl I Arslan Z. An unusual presentation of Kawasaki disease: gallbladder hydrops and acute cholestatic hepatitis. Case Rep Med. (2018) 2018:4930234. 10.1155/2018/4930234 30057618 PMC6051122

[10] Anjani G Deglurkar R Pilania R Chaudhary H Vaiphei K Vignesh P. Fulminant acute liver failure as an unusual presentation of Kawasaki disease. Scand J Rheumatol. (2021) 50:327–9. 10.1080/03009742.2020.1812711 33205690

[11] Morita A Imagawa K Ishiodori T Tagawa M Takada H. Kawasaki disease with dilatation of the common bile duct: A case report and review of literature. Int J Rheum Dis. (2021) 24:1325–30. 10.1111/1756-185X.14208 34424609

[12] Menon J Shanmugam N Vasudevan A Kumar N Rammohan A Rela M. Kawasaki disease in a pediatric liver transplant patient. Transplant Immunology. (2021) 67:101416. 10.1016/j.trim.2021.101416 34033866

[13] Paglia P Nazzaro L Anseris D Elena AG Lettieri M Colantuono R. Atypically protracted course of liver involvement in Kawasaki disease. Case Report and Literature Review Pediatric Reports. (2021) 13:357–62. 10.3390/pediatric13030044 34287369 PMC8293418

[14] Sarkar S Bhattacharyya B Agarwal A. Jaundice at the onset: A rare event in Kawasaki disease. Indian J Pediatr. (2021) 88:379–80. 10.1007/s12098-020-03559-7 33175362

[15] Bylund W Zarow GJ Ponce DM. Acute jaundice in a six-year-old: an unusual presentation of atypical Kawasaki disease. Clin Pract Cases Emerg Med. (2020) 4:142–5. 10.5811/cpcem.2019.12.45180 32426656 PMC7219998

[16] Martínez Vázquez JA Sánchez García C Rodríguez Muñoz L Martínez Ramírez RO. Acute kidney injury and cholestasis associated with Kawasaki disease in a 9-year-old: Case report. Reumatol Clin (Engl Ed). (2019) 15:e114–5. 10.1016/j.reumae.2017.11.003 29254742

[17] Koca T Aslan N Akaslan Kara A Pektas A Ozen M Akcam M. Kawasaki disease in a 9-year old girl presenting with febrile cholestasis: case report and review of literature. Int J Rheum Dis. (2018) 21:2046–9. 10.1111/1756-185X.12700 26177575

[18] Goknar N Demir AD Ataman Y Gokalp S Oktem F Kasapcopur O. case of Kawasaki disease with initial presentation of arthritis and icterus. Bezmialem sci. (2017) 5:86–9. 10.14235/bs.2016.939

[19] Kaman A Aydin-Teke T Gayretli-Aydin ZG Öz FN Metin-Akcan Ö Eriş D. Two cases of Kawasaki disease presented with acute febrile jaundice. Turk J Pediatr. (2017) 59:84–6. 10.24953/turkjped.2017.01.015 29168370

[20] Keeling IM Beran E Dapunt OE. Kawasaki disease and hepatobiliary involvement: report of two cases. Ital J Pediatr. (2016) 42:1–3. 10.1186/s13052-016-0238-7 26951087 PMC4782281

[21] Rosencrantz RA Huang T Sonke PY Tewari D Chander PN. Autoimmune sclerosing cholangitis: An atypical association with kawasaki disease. Hepatology. (2016) 64:2253–6. 10.1002/hep.28694 27333792

[22] Perera PJ Samarasinghe D Pathirana D Randeni S Samdamal LYS. An atypical case of Kawasaki disease presenting with cholestatic jaundice. Sri Lanka J Child Health. (2015) 44:58–60. 10.4038/sljch.v44i1.796532051639

[23] Talebian A Soltani B Rezaei MH. Jaundice as an unusual presentation of Kawasaki disease: A case report. Arch Pediatr Infect Dis. (2015) 3:e27594. 10.5812/pedinfect.27594

[24] Grewal A Singh S Suri D Lal S Manojkumar R Thapa BR. Kawasaki disease masquerading as jaundice. Indian J Pediatr. (2013) 80:261–2. 10.1007/s12098-012-0736-6 22447618

[25] Jafari SA Kiani MA Ahanchian H Khakshour A Partovi S Kianifar HR. Kawasaki disease presenting as acute clinical hepatitis. Int J Pediatr. (2013) 1:51–3.

[26] Karpathios T Moustaki M Yiallouros P Sharifi F Attilakos A Papadopoulou A. Severe jaundice in two children with Kawasaki disease: a possible association with Gilbert syndrome. J Korean med sci. (2012) 27:101–3. 10.3346/jkms.2012.27.1.101 22219623 PMC3247765

[27] Ibáñez-Alcalde M Sánchez-Forte M Giménez-Sánchez F Ortega-Montes Á Martínez-Espinosa G. Cholestasis as the initial feature of Kawasaki disease. Pediatr Infect Dis J. (2012) 31:766–7. 10.1097/INF.0b013e318253a1d8 22426301

[28] Taddio A Pellegrin MC Centenari C Filippeschi IP Ventura A Maggiore G. Acute febrile cholestatic jaundice in children: keep in mind Kawasaki disease. J Pediatr Gastroenterol Nutr. (2012) 55:380–3. 10.1097/MPG.0b013e31825513de 22437475

[29] Grech V Buttigieg T Portelli A Kotes S Attard G Huhn P. Kawasaki disease presenting as hepatitis. Ann Trop Paediatr. (2007) 27:303–6. 10.1179/146532807X245706 18053348

[30] Chen WT Huang SR Wang JK. Kawasaki disease presenting with hepatitis and prolonged fever: report of one case. Acta Paediatrica Taiwanica. (2003) 44:174–6. 14521027

[31] Bal AK Prasad D Umali Pamintuan MA Mammen-Prasad E Petrova A. Timing of intravenous immunoglobulin treatment and risk of coronary artery abnormalities in children with Kawasaki disease. Pediatr Neonatol. (2014) 55:387–92. 10.1016/j.pedneo.2013.11.007 24636168

[32] Rife E Gedalia A. Kawasaki Disease: an Update. Curr Rheumatol Rep. (2020) 22:75. 10.1007/s11926-020-00941-4 32924089 PMC7487199

[33] Lee MN Cha JH Ahn HM Yoo JH Kim HS Sohn S. Mycoplasma pneumoniae infection in patients with Kawasaki disease. Korean J Pediatr. (2011) 54:123–7. 10.3345/kjp.2011.54.3.123 21738542 PMC3120998

[34] Tang Y Yan W Sun L Huang J Qian W Hou M. Kawasaki disease associated with Mycoplasma pneumoniae. Ital J Pediatr. (2016) 42:83. 10.1186/s13052-016-0292-1 27609267 PMC5016862

[35] Vitale EA La Torre F Calcagno G Infricciori G Fede C Conti G. Mycoplasma pneumoniae: a possible trigger of Kawasaki disease or a mere coincidental association? Report of the first four Italian cases. Minerva Pediatr. (2010) 62:605–7. 21042274

[36] Narita M. “Mycoplasma pneumoniae as an under-recognized agent of vasculitic disorders,” in Advances in the Etiology, Pathogenesis and Pathology of Vasculitis, ed Amezcua-Guerra L. M. (Rijeka: In Tech). (2011) 37–56. Available online at: http://www.intechopen.com/books/advances-in-the-etiologypathogenesis-and pathology-of-vasculitis/mycoplasma-pneumoniae-as-anunder-recognized-agent-of-vasculitic disorders. 10.5772/22875

[37] Narita M. Classification of Extrapulmonary Manifestations Due to Mycoplasma pneumoniae Infection on the Basis of Possible Pathogenesis. Front Microbiol. (2016) 7:23. 10.3389/fmicb.2016.00023 26858701 PMC4729911

[38] Poddighe D. Mycoplasma pneumoniae-related hepatitis in children. Microb Pathog. (2020) 139:103863. 10.1016/j.micpath.2019.103863 31712120

